# Cosplicing network analysis of mammalian brain RNA-Seq data utilizing WGCNA and Mantel correlations

**DOI:** 10.3389/fgene.2015.00174

**Published:** 2015-05-13

**Authors:** Ovidiu D. Iancu, Alexandre Colville, Denesa Oberbeck, Priscila Darakjian, Shannon K. McWeeney, Robert Hitzemann

**Affiliations:** ^1^Department of Behavioral Neuroscience, Oregon Health & Science UniversityPortland, OR, USA; ^2^Division of Biostatistics, Public Health and Preventative Medicine, Oregon Health & Science UniversityPortland, OR, USA; ^3^Research Service, Veterans Affairs Medical CenterPortland, OR, USA

**Keywords:** gene cosplicing, scale-free gene networks, brain transcriptome, alternative splicing, gene coexpression

## Abstract

Across species and tissues and especially in the mammalian brain, production of gene isoforms is widespread. While gene expression coordination has been previously described as a scale-free coexpression network, the properties of transcriptome-wide isoform production coordination have been less studied. Here we evaluate the system-level properties of cosplicing in mouse, macaque, and human brain gene expression data using a novel network inference procedure. Genes are represented as vectors/lists of exon counts and distance measures sensitive to exon inclusion rates quantifies differences across samples. For all gene pairs, distance matrices are correlated across samples, resulting in cosplicing or cotranscriptional network matrices. We show that networks including cosplicing information are scale-free and distinct from coexpression. In the networks capturing cosplicing we find a set of novel hubs with unique characteristics distinguishing them from coexpression hubs: heavy representation in neurobiological functional pathways, strong overlap with markers of neurons and neuroglia, long coding lengths, and high number of both exons and annotated transcripts. Further, the cosplicing hubs are enriched in genes associated with autism spectrum disorders. Cosplicing hub homologs across eukaryotes show dramatically increasing intronic lengths but stable coding region lengths. Shared transcription factor binding sites increase coexpression but not cosplicing; the reverse is true for splicing-factor binding sites. Genes with protein-protein interactions have strong coexpression and cosplicing. Additional factors affecting the networks include shared microRNA binding sites, spatial colocalization within the striatum, and sharing a chromosomal folding domain. Cosplicing network patterns remain relatively stable across species.

## Introduction

There are several different strategies that can be used to analyze gene expression data (Allen et al., 2012; Jay et al., 2012). Systems biology approaches such as the Weighted Gene Coexpression Network Analysis (WGCNA) have shown that the coexpression structure follows a power-law distribution, clusters the expression data into modules of conserved function which are preserved to varying degrees across cell types and species and importantly allows one to detect patterns of gene connectivity that can be aligned with behavioral and physiological phenotypes (e.g., Zhou et al., 2011; Konopka et al., 2012). The emphasis on gene connectivity frequently focuses on detecting highly connected “hub” genes that are potential targets for therapeutic manipulation. Although methods such as the WGCNA have been widely used for the analysis of microarray data, they have been only recently applied to RNA-Seq data, which not only has improved clustering metrics but also provides exon-level resolution (Iancu et al., 2012b; Giorgi et al., 2013). Recent studies (Chen and Zheng, 2009; Dai et al., 2012) have shown that these exon data can be used to generate cosplicing networks that are distinct in structure and function from the coexpression networks. These studies have demonstrated that individual exons from different genes can have correlated expression levels even when no correlation is detectable between the overall gene expression levels.

The current study investigates the structure of the cosplicing networks by implementing an analysis strategy that involves the construction of distance (similarity/dissimilarity) matrices. Previously we have used distance-based approaches to examine high-dimensional genotype data for the purpose of illustrating how selective breeding affects allele segregation (Iancu et al., 2010, 2012a, 2013a,b). We note that a preliminary outline of results presented here has been previously published in Iancu et al. (2014). Other examples of using distance measures to analyze genotype and gene expression data abound (e.g., Zapala and Schork, 2006, 2012). We now extend the distance approach to exon level gene expression data. Gene transcripts are represented as a list/vector of exon expression levels, which for RNA-Seq datasets are proportional to the exon level read counts. Many genes have the potential for alternative splicing, and therefore gene-level expression reflects a collection of isoforms; relative abundance of isoforms translates into relative exon inclusion rates. It is often unclear whether changes in “expression level” originate from one or more isoforms. Given this data representation, an ecological analysis strategy (Mantel, 1967) was used to correlate exon inclusion rates across samples. The major benefit of this approach rested in avoiding the representation of a gene (a complex mixture of isoforms, each consisting of multiple exons) as a single scalar value; such a limited approach, unavoidable in the case of gene microarray data, is sub-optimal when exon data are available. To capture cosplicing events, we utilized two distinct distance measures. The cosine distance measure, which is not affected by changes in overall gene expression level, has been previously utilized for alternative splicing detection (Aschoff et al., 2013). We utilized this measure for the construction of the cosplicing network. Additionally, we also utilized the Canberra distance measure, which is sensitive to changes in overall gene expression levels as well as exon inclusion patterns; this distance measure generated a distinct network which we denote as CoSplicEx. Gene networks were then constructed using the WGCNA approach, using the Mantel correlations to derive network edge weights. We found that the resulting cosplicing and CoSplicEx network structures are clearly distinct from the structure of the gene coexpression networks derived from the same data, both in terms of network topology and in terms of the biological factors driving the network structure.

## Materials and methods

### RNA-Seq data collection and pre-processing

The RNA-Seq data consisted of a total of 60 animals that are the result of previous generations of selective breeding for haloperidol response (Iancu et al., 2012a). Tissue collection and preparation was performed as described previously (Iancu et al., 2012a). The reads were aligned using the Bowtie short read alignment program (Langmead et al., 2009) to the reference mouse genome (NCBI m37 assembly) and summarized relative to Ensembl 59 gene models using the “union exon” framework. The gene and exon counts were corrected using edgeR (Robinson et al., 2010) upper quartile normalization factors. Data is publicly available as GEO series GSE62669.

We employed additional procedures to mitigate possible deleterious effects of low/noisy exon counts. First, we removed from the analysis genes with less than 500 average counts. Next, we constructed a large pairwise adjacency matrix of all exon pairwise Pearson correlations. Based on this matrix we computed the network connectivity of all exons and removed from network construction genes where all exons have low (bottom quartile) network connectivity. After these selection procedures we retained 9066 genes for network construction.

### Network construction methodology

Construction and annotation of the transcriptional networks was performed largely as described elsewhere (Langfelder and Horvath, 2008; Iancu et al., 2010); for the cosplicing network we used the Mantel correlation as detailed below. Gene coexpression is often quantified using correlation of gene expression levels. When a gene is represented as a vector of exon counts, we compute the pairwise distances between the N samples, resulting in an N by N matrix that contains N(N-1)/2 unique distances. The choice of distance measure reflects the goals of the analysis. For the CoSplicEx and cosplicing networks we used the Canberra and cosine distance measures, respectively:

$$d_{A}^{C}\left(i,j\right)=\sum_{e\;=\;1}^{p}\frac{\left|a_{e}^{i}-a_{e}^{j}\right|}{\left|a_{e}^{i}+a_{e}^{j}\right|},d_{A}^{Cos}\left(i,j\right)=1-\frac{{\vec{a}}^{i}\cdot {\vec{a}}^{j}}{\left\|{\vec{a}}^{i}\right\|\cdot \left\|{\vec{a}}^{j}\right\|}$$

where *i*, *j* are two samples and e iterates over all exon counts a. In the Canberra metric each exon contributes a value between 0 and 1 to the total distance.

### Comparison and annotation of network hubs

The number of exons, annotated transcripts, protein domains, coding sequence size, homology information and GC percentage content were retrieved using the biomaRt R package (Durinck et al., 2009). Gene markers for mouse neuronal cell types were retrieved from Cahoy et al. (2008). The statistical comparisons between hub characteristics were performed using Wilcoxon rank-sum test available in R.

### Quantification of factors affecting network edges

Information about known protein-protein interactions (PPI) in the mouse was gathered from the BioGRID database (Chatr-Aryamontri et al., 2013). Predicted transcription factor binding sites (TFBS) for each gene in the network were acquired using the Promoter Analysis and Interaction Network Tool (PAINT) (Vadigepalli et al., 2003), which uses the TRANSFAC database (Matys et al., 2003). To compare the identity of TFBS between any two genes, we used the Jaccard distance measure: two genes were deemed to have high overlap of TFBS if the Jaccard measure was >0.5, meaning that the number of TFBS in the intersection was at least half of all the TFBS detected in the two genes.

Computationally predicted splicing factor binding sites were acquired from the SFMap database (Akerman et al., 2009; Paz et al., 2010). First, we retrieved from biomaRt the genomic coordinates of all genes in our networks. The genomic coordinates were used as input to the SFMap algorithm, using the default settings. The SFMap database returned information about the following 21 splicing factors: *SF2ASF, 9G8, SC35, Tra2alpha, Tra2beta, SRp20, SRp40, SRp55, hnRNPA1, hnRN-PA2B1, hnRNPF, hnRNPH1, hnRNPM, hnRNPU, MBNL, NOVA1, PTB, CUG-BP, YB1, FOX1, QK1*. Next, we devised a procedure to compare the number and identity of the SFBS for any pair of genes. To represent both the identity and multiplicity of each SFBS for each gene, we constructed vectors of length 21 with entries represented by the multiplicity of each SFBS within the gene. For each pair of genes, we constructed Canberra distances based on these SFBS vectors. The distances varied between 0 and 21, the lower quartile (<7.6) was selected to represent gene pairs with high overlap in terms of SFBS. Predicted interactions between genes and microRNAs were identified using the microRNA R package (Gentleman and Falcon)^1^; we compared correlations between genes sharing at least two microRNA binding sites against correlations between random pairs of genes.

Chromosomal topological domain boundary from the mouse cortex was retrieved from Dixon et al. (2012). Here, we compared pairs of genes that were located within the chromosomal folding domain against pairs of genes that were located within the same range of base pair distances along the chromosome, but were nevertheless located on different folding domains. Spatial gene co-localization was retrieved from the Allen Brain Atlas using the NeuroBlast algorithm (Ng et al., 2007); this algorithm returns, for each gene, the top 250 genes most similar in spatial distribution within a brain region (in our case the striatum). We compared network correlations between spatially similar genes vs. random groups of genes.

Functional significance of modules and other genes groups of interest was evaluated using Gene Ontology (GO) enrichment analysis (Ashburner et al., 2000) using the GO-stats R package (Falcon and Gentleman, 2007). To correct for the nested structure of the GO terms, we utilized a graph decorrelation procedure that limits the GO annotations to the most specific terms (Alexa et al., 2006). The background group for all GO analyses was the 9066 genes included in network construction.

## Results

### Coexpression, cosplicing, and CoSplicEx network construction

The test dataset was new RNA-Seq (polyA+) data obtained from the striatum of heterogeneous stock-collaborative cross (HS-CC) mice; *N* = 60 and approximately 30 million reads were obtained per sample, unique alignment to the reference genome was > 85% and the upper quartile was used for normalization as in Bottomly et al. (2011). The HS-CC was formed from the eight inbred strains used to generate the collaborative cross (Churchill et al., 2004); the CC strains include three wild-derived strains and appear to encompass approximately 90% of the genetic diversity available in *Mus musculus* (Roberts et al., 2007).

**Figure 1:**
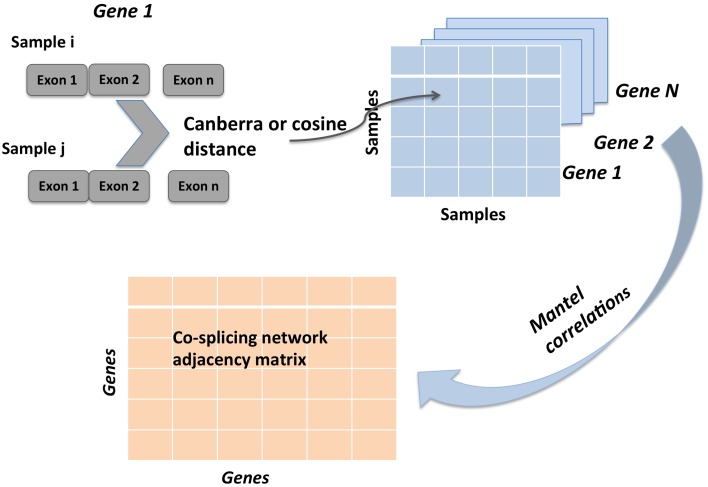
**Illustration of cosplicing network construction steps**. RNA-Seq data is summarized as read counts mapped to individual exons. For each gene, pairwise distance between all samples is computed. This first step results in N (one for each gene) square matrices of size equal to number of samples. Second, Mantel correlations between all N gene matrices are computed, resulting in the cosplicing network matrix.

**Figure 2:**
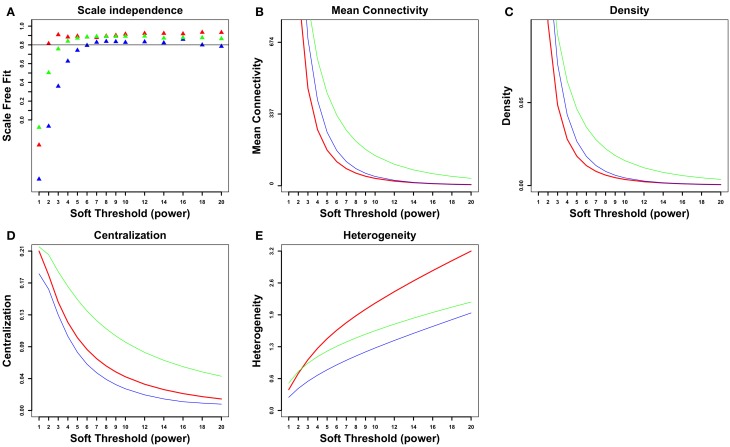
**Network properties of coexpression (blue), cosplicing (green) and CoSplicEx (red) networks. (A)** Scale independence, **(B)** Mean connectivity, **(C)** Mean density, **(D)** Centralization, **(E)** Heterogeneity.

A gene coexpression network was constructed as described elsewhere (Iancu et al., 2013a) using the Pearson correlation to form the adjacency matrix, following the WGCNA framework (Langfelder and Horvath, 2008). A total of 9066 genes were entered into the analysis. The cosplicing and CoSplicEx network construction procedure followed the same general approach as for coexpression but using a different type of correlation that incorporated information about differences in exon usage. First, each gene was represented as a vector/list of exon vector counts; pairwise differences between all available sample pairs were computed using the cosine or Canberra distance measure, respectively. Second, the distance matrices were correlated using the Mantel procedure. An outline of the cosplicing and CoSplicEx network construction procedure is illustrated in Figure 1. Subsequent steps in the construction of the cosplicing and coexpression networks were identical. Network edge weight in the adjacency matrix was computed by raising the correlation to a power β. All three networks were scale free with connectivity having an exponential distribution (Figure 2A). As the value of β was increased, all networks converged to a scale-free structure (Zhang and Horvath, 2005) with the CoSplicEx network displaying faster convergence (Figure 2A); however, to facilitate comparison of the network properties *β* = 6 was used uniformly in all calculations. Network heterogeneity, centrality, and density for all networks were calculated as defined in Langfelder and Horvath (2008). Heterogeneity and centrality were significantly higher for the CoSplicEx network but density was higher for the coexpression network (Figures 2B–E). Biologically, these results suggest that coexpression is more widespread (high density) while the CoSplicEx network is characterized by fewer but stronger interactions (higher centrality).

### Comparison of cosplicing, CoSplicEx, and coexpression hubs

**Figure 3:**
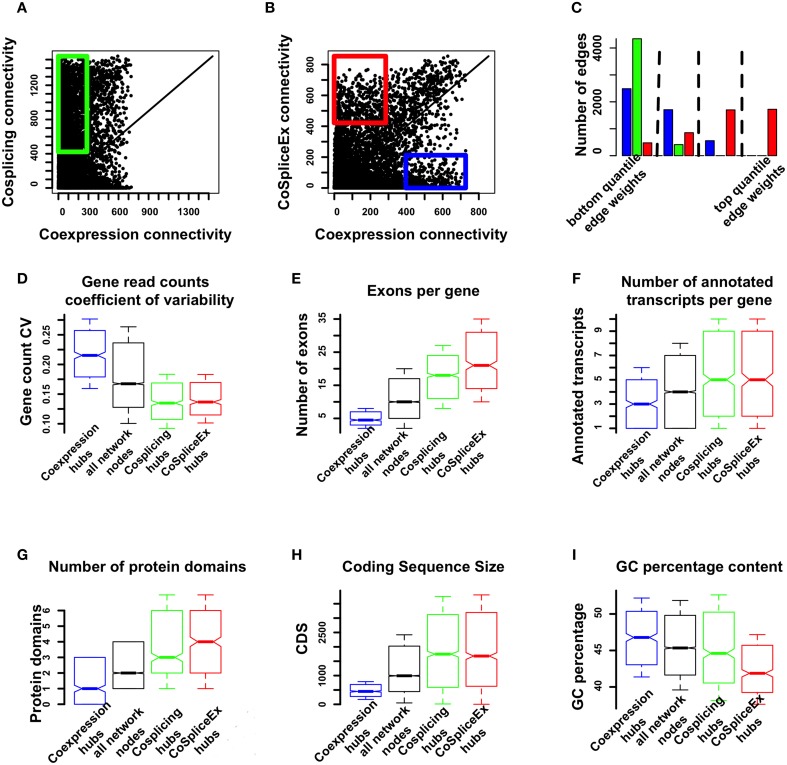
**Comparison of coexpression and cosplicing connectivity. (A)** Connectivity comparison, cosplicing vs. coexpression. Green rectangle: nodes in top 10% cosplicing connectivity, bottom 80% coexpression connectivity. **(B)** Connectivity comparison, coexpression vs. CoSplicEx networks. Blue rectangle: nodes in top 10% coexpression connectivity, bottom 80% CoSplicEx connectivity. Red rectangle: nodes in top 10% CoSplicEx connectivity, bottom 80% coexpression connectivity. **(C)** Distribution of edges between exclusive coexpression and cosplicing hubs. Only the CoSplicEx network detects strong edges between these hubs. **(D)** Coefficient of variation for total gene counts. **(E)** Number of exons per gene. **(F)** Number of annotated transcripts per gene. **(G)** Number of protein domains. **(H)** Coding sequence size. **(I)** GC percentage content.

To evaluate whether the three networks provide distinct information, the node connectivity distributions were compared. In particular we focus on the coexpression and CoSplicEx networks in order to identify network features that arise from the fact that CoSplicEx is sensitive to cosplicing in addition to coexpression. We selected from the coexpression and CoSplicEx networks hub genes (top 10% connectivity) that were specific to these networks and had low (bottom 80%) coexpression connectivity. These hubs are outlined in green (Figure 3A) or red (Figure 3B), respectively. Similarly, we selected a set of coexpression hubs that had low CoSplicEx connectivity (outlined in blue in Figure 3B). These unique coexpression (*N* = 497), CoSplicEx (*N* = 425), and cosplicing (*N* = 495) could be distinguished by several characteristics. The cosplicing and CoSplicEx hubs had a lower gene count coefficient of variability (Figure 3D), due to the fact that they were selected among the genes with low coexpression connectivity. The CoSplicEx and cosplicing hubs had a higher average number of exons and annotated transcripts (Figures 3E,F), more protein domains, longer coding size and lower GC content (Figures 3G–I).

The coexpression and cosplicing networks are designed capture specific information about coordination in the expression and splicing profile of gene pairs, respectively. However, there is an additional mechanism by which genes could be co-regulated: changes in the expression level of one gene could be related to changes in the splicing profile of other genes. The CoSplicEx network is designed to capture this type of interaction. To illustrate the capacity of the CoSplicEx network to capture expression-splicing interactions, we selected two sets of highly exclusive coexpression and cosplicing hubs. The first set of genes had coexpression connectivity in top 10%, while cosplicing connectivity was in the bottom 10%. Conversely, a second set of genes had high cosplicing but low coexpression connectivity. Next, we examined the edge strengths between these distinct hub genes in all three networks. The total set of edges in each network was divided into quartiles and the edges between hub types were mapped onto these quartiles. As illustrated in Figure 3C, only the CoSplicEx network contains strong edges between coexpression and cosplicing hubs.

The coexpression and CoSplicEx hubs were aligned with known markers of neurons and neuroglia (Cahoy et al., 2008). The CoSplicEx hubs were over-represented among neurons, astrocytes and oligodendrocytes (Fisher's exact test *p* < 2 × 10^−5^, 0.03 and 4 × 10^−8^, respectively), while the coexpression hubs were under-represented (*p* < 5 × 10^−11^, 0.01 and 0.004, respectively).

GO annotation of the CoSplicEx hubs revealed enrichment in neurogenesis (*p* < 2 × 10^−9^), neuron projection (*p* < 10^−11^), enzyme binding (*p* <3 × 10^−7^) (Supplemental Table 1). Annotation of the coexpression hubs revealed enrichment in electron transport chain (*p* <10^−19^), mitochondrial part (*p* <2×10^−23^) and hydrogen ion transmembrane transporter activity (*p* <3×10^−12^) (Supplemental Table 1). Completely absent from the coexpression annotation, over a wide range of enrichment scores, were terms related to neuronal function. As also seen in Figure 3A, there was a group of genes exhibiting a mixture of high coexpression and CoSplicEx connectivity, here defined as the top 20% in both categories (but excluding the hubs described above). Annotation of these hubs revealed enrichment in chromatin modification (*p*<2 × 10^−7^), neurogenesis (*p*<4 × 10^−7^) and brain development (*p*<10^−5^) (Supplemental Table 1).

### Evolutionary history of network hubs

**Figure 4:**
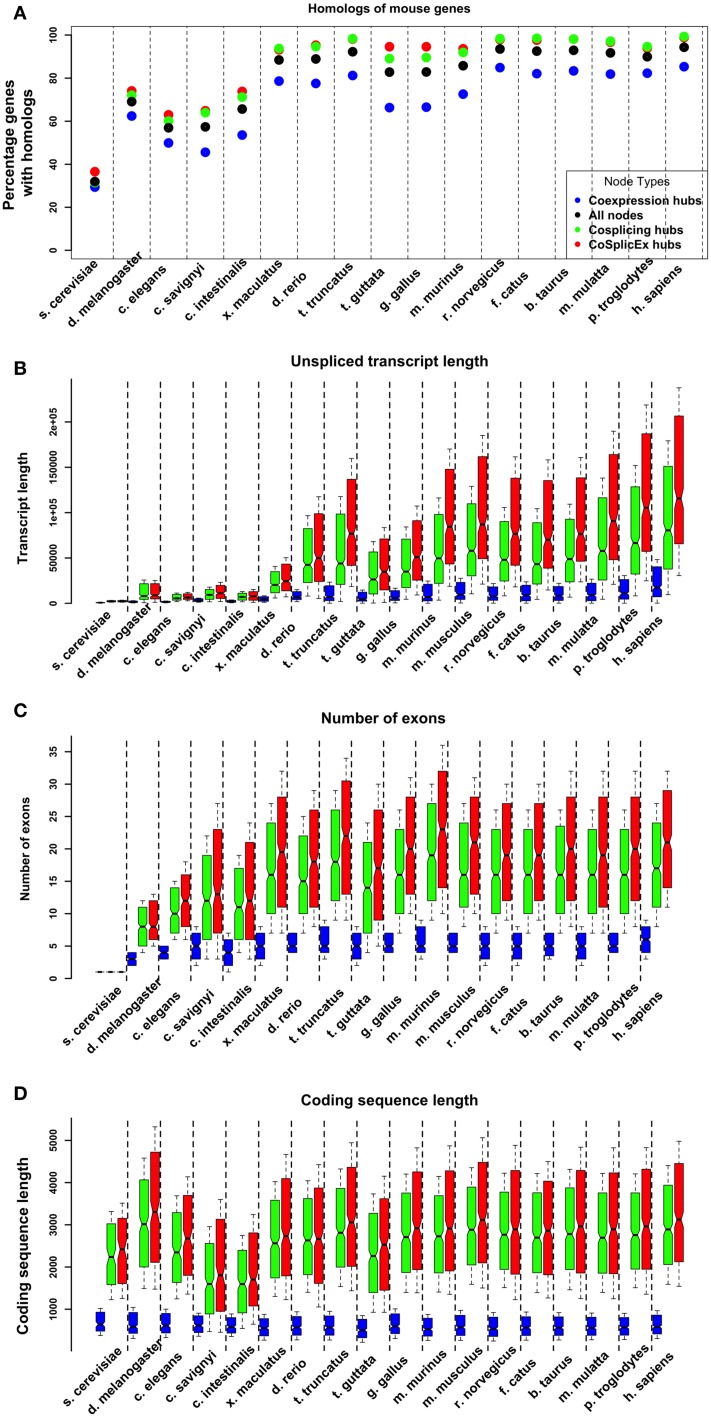
**Evolutionary properties of coexpression (blue) and cosplicing (red) network hubs. (A)** Percentage of hub genes with homologs across species from yeast to human. **(B)** Length (base pairs) of longest unspliced transcript. **(C)** Number of exons in longest transcript. **(D)** Coding region length of longest transcript.

The characteristics of the gene homologs corresponding to the network hubs were examined. Across all organisms, the cosplicing, and CoSplicEx hubs had higher proportion of homologs (Figure 4A). These homologs had higher number of exons, longer un-spliced transcript length and longer coding region length. Comparing cosplicing homolog characteristics across organisms from yeast to human, large increases were detected in the number of exons and lengths of un-spliced transcripts; however, the length of the coding region was preserved (Figures 4B–D).

### Factors affecting edge weights

**Figure 5:**
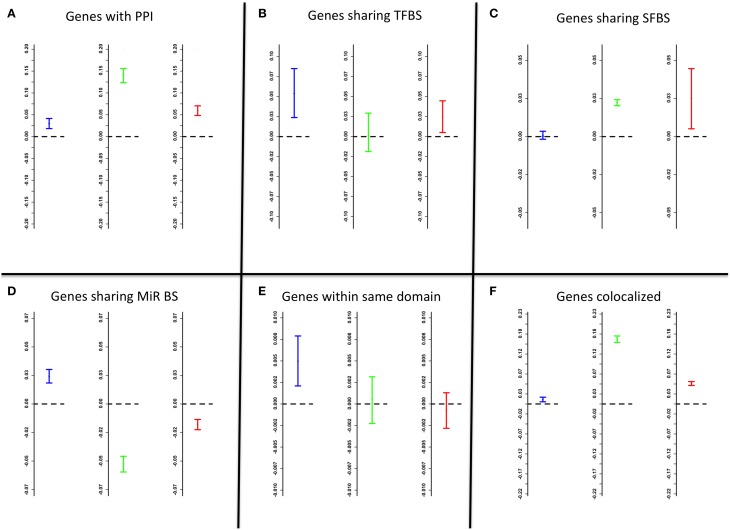
**Factors affecting edge weights**. Data represented as 95% confidence intervals; intervals above/bellow the zero (dashed) line indicate significant strengthening/weakening of edges. Blue, green, and red confidence intervals correspond to coexpression, cosplicing, and CoSplicEx edges, respectively. **(A)** genes with known protein-protein interactions. **(B)** genes sharing transcription factor binding sites. **(C)** genes sharing splicing factor binding sites. **(D)** genes sharing microRNA binding sites. **(E)** genes in the same topological chromosomal domain. **(F)** genes colocalized.

The following factors were evaluated for association with the network edge weights: known PPI, shared TFBS, shared splicing factor binding sites (SFBS), shared microRNA binding sites, shared chromosomal folding topological domains and spatial co-localization within the striatum. In both networks, for genes with known PPI the edge weights were significantly higher (Mann-Whitney test average location shift mu = 0.03 for coexpression, mu = 0.14 for cosplicing, and mu = 0.06 for CoSplicEx, *p* < 10^−15^) than for random gene pairs (Figure 5A). Gene pairs sharing TFBS had increased coexpression and CoSplicEx edges (mu = 0.06, *p* < 3 × 10^−5^), but not cosplicing (Figure 5B). The reverse was true for SFBS (Figure 5C): gene pairs sharing SFBS had stronger cosplicing and CoSplicEx (mu = 0.028 and mu = 0.03 respectively, *p* < 10^−15^) but coexpression was only modestly increased. Importantly, it was observed that CoSplicEx and cosplicing connectivity had a modest but statistically significant correlation with the number of SFBS within a gene (*r* = 0.22 and *r* = 0.15 respectively, *p* < 10^−15^).

For genes sharing microRNA binding sites, coexpression was modestly increased but cosplicing and CoSplicEx were significantly decreased (*p* < 10^−15^) (Figure 5D). Genes sharing topological chromosomal folding domains (Dixon et al., 2012) displayed increased coexpression (*p* < 10^−15^) but not increased cosplicing or CoSplicEx connectivity (Figure 5E). It was previously observed (Iancu et al., 2010) that striatal gene coexpression is strengthened for genes with spatially overlapping patterns of gene expression (Ng et al., 2007). Colocalization increased edge strengths in all networks (*p* < 10^−15^) (Figure 5F).

### Characterization of modules

**Figure 6:**
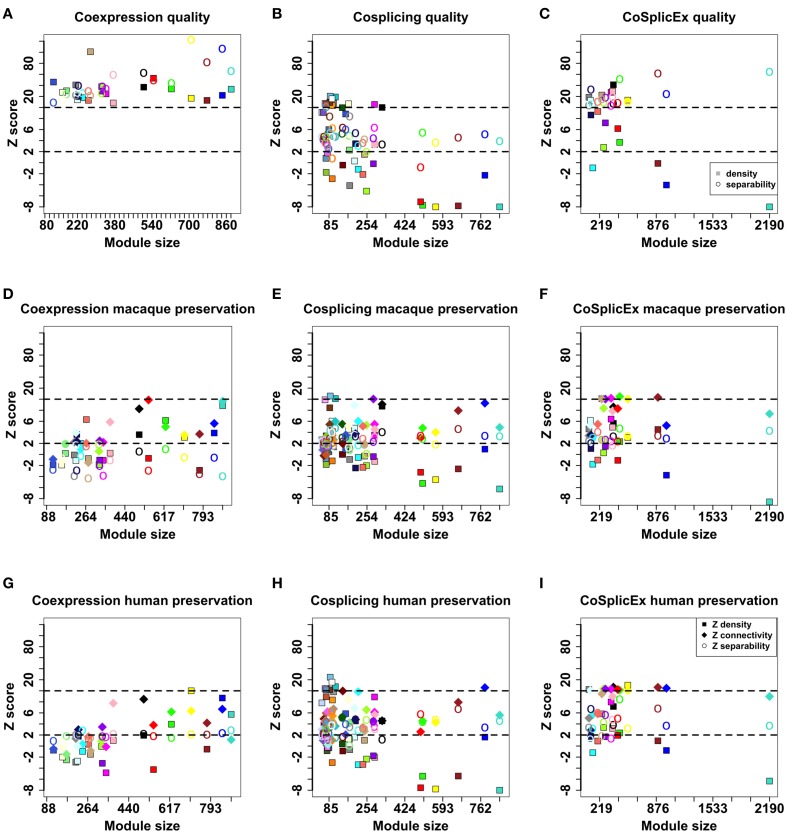
**Module quality values in the coexpression **(A)**, cosplicing **(B)**, and CoSplicEx **(C)** networks**. Module preservation values between mouse striatum and macaque cortex for coexpression **(D)**, cosplicing **(E)**, and CoSplicEx **(F)** networks. Module preservation values between mouse striatum and human cortex for coexpression **(G)**, cosplicing **(H)**, and CoSplicEx **(I)** networks.

The three networks were clustered into modules as described in Langfelder et al. (2008); modules are denoted by arbitrary colors independently assigned in each network. To evaluate the performance of module detection, different types of module quality measures were computed: overall quality, connectivity, density, and separability. Detailed definitions of these concepts are available in Langfelder et al. (2011). Briefly, density preservation implies that network hubs remain highly connected across the networks compared. Separability measures whether genes assigned to modules/clusters are indeed more connected to each other than to genes outside the module. All network measurements were expressed as Z scores; Z scores < 2 were taken to imply poor module quality. It was observed that all modules in the coexpression network had overall high quality (*Z* > 10); while in the cosplicing network density quality measures were relatively low, with CoSplicEx quality good except for four of the density module quality numbers (Figures 6A–C).

### Module preservation across species

Coexpression and cosplicing module preservation was investigated using RNA-Seq data obtained from the rhesus macaque ventral-medial prefrontal cortex (32 samples) and from human prefrontal cortex (30 samples). Network measurements were expressed as Z scores; Z scores < 2 were taken to imply poor module preservation, while higher values were taken to signify moderate (>2) or high (>10) module preservation. In addition to measures of module density and separability described above, we evaluated connectivity preservation which evaluates whether the patterns of connections between groups of genes remains unaltered. These measures were computed for all modules and are presented in Figures 6D–I. Generally, connectivity, and separability were more preserved than density measures. Cosplicing and CoSplicEx modules were more preserved across species than coexpression modules, even though the latter had better quality measures in the mouse network. A larger percentage of homologs for the cosplicing and CoSplicEx hubs probably contributes to better module preservation values.

## Discussion

The advantages and disadvantages of RNA-Seq compared to microarrays to analyze the brain transcriptome have been discussed elsewhere (e.g., Hitzemann et al., 2013); one of the important advantages is that RNA-Seq provides detailed information on alternative exon usage which is unusually high in the brain, especially during brain development (Johnson et al., 2009). The precise number of transcripts expressed in brain is unknown but may well-number in the hundreds of thousands, the transcripts are likely to differ markedly among brain regions and cell types and minor transcripts in terms of abundance may have significant biological importance. Further, although a fair amount is known about how splicing factors can coordinate exon inclusion/exclusion for relatively small gene clusters (e.g., Ule et al., 2005), the regulation of splicing on a genome-wide basis may well-involve additional mechanisms/interactions. Kelemen et al. (2013) have summarized the data which points to the global regulation of alternative splicing. In addition to development, coordinated changes in splicing have been observed during myogeneic differentiation (Bland et al., 2010), differentiation of human erythrocyte (Yamamoto et al., 2009), during epithelial mesenchymal transition (Warzecha et al., 2010), during chemically induced cell death (Moore et al., 2010), during cancer metastasis (Lu et al., 2015) and after insulin stimulation (Hartmann et al., 2009). In addition, alternative splicing shows distinct differences between human and chimpanzees brains (Calarco et al., 2007).

There are two general approaches for quantifying gene expression using RNA-Seq data. The first approach attempts to probabilistically quantify the expression of each different gene isoform, with subsequent analysis steps applied to the estimated isoform expression levels—for a review see for example (Garber et al., 2011). An alternative approach, which is not dependent on a priori knowledge of the identity of the isoforms and the potentially unreliable estimation of their expression levels, evaluates all exon expression values for a given gene simultaneously, using a distance measure approach (Aschoff et al., 2013). We employ here the same general distance-measure based strategy, but instead of focusing on differential splicing we use the computation of the distance measures as an intermediate step toward constructing a cosplicing network. The use of distance measures offers a set of desirable characteristics. First, using distances allows a transcriptome level evaluation of correlation patterns between hundreds of thousands of exons with only moderate computational load. Second, even though exon level data is used to infer the network, this information is integrated at the gene level. This facilitates integration and summarization, since the unit of biological activity is more often the gene and not the individual exon, with annotation databases reflecting this reality. Finally, because genes remain the nodes of the cosplicing network, a direct transcriptome wide comparison between gene coexpression and gene cosplicing network properties is feasible.

Approximately 5% of the genes entered into our analyses were identified as highly connected coexpression or cosplicing hubs unique to each network (Figure 3A); we also identified a population of genes that were both highly coexpressed and cospliced and appeared functionally distinct from the unique coexpression and cosplicing hubs. Characterization of the coexpression, cosplicing, and CoSplicEx hubs revealed some striking differences. Not unexpectedly, the cosplicing and CoSplicEx hubs were longer, more complex genes with more exons, with more annotated transcripts and with greater variability in exon inclusion rates. The coexpression hubs were mainly involved in ubiquitous cell processes such as metabolism and energy production; in contrast the CoSplicEx and cosplicing hubs were highly annotated with terms related to neuronal function e.g., the synapse. To some extent, this apparent biological specialization of the hub types is reflected in the fact that there is no correlation between coexpression and CoSplicEx or cosplicing connectivity (Figures 3A,B). The coexpression hubs appeared different between the cosplicing hubs in terms of GC content as well, with CoSplicEx and cosplicing hubs exhibiting significantly lower GC content. This relationship held for all hub homologs (data not shown). Lower GC content in these hubs is caused by the long intronic sequences with low GC content, which has been shown to be an important factor in splice site recognition (Amit et al., 2012). Taken together, these biological and computational observations suggest that coexpression and cosplicing serve distinct roles within the transcriptional system, consistent with findings across a variety of systems (Calarco et al., 2007; Stilling et al., 2014; Lu et al., 2015).

King et al. (2013) have observed that a topoisomerase one inhibitor (topotecan), dose-dependently reduced the expression of very long genes in mouse and human neurons. Further, these authors noted that a significant percentage of the reduced expression genes were autism spectrum disorder (ASD) candidate genes. As part of this analysis (Zhou et al., 2011) tabulated from several sources a list of 974 ASD candidates. Of these 974 candidates, 660 were entered into our network analysis. 65 of these ASD genes are also among the 425 unique CoSplicEx hubs; this represents a significant enrichment (*p* < 2 × 10^−8^). These data illustrate that the CoSplicEx hubs may have a significant role in neurodevelopmental disorders. In contrast, only 20 of the unique 497 coexpression hubs were ASD candidates; this represented a modestly significant enrichment (*p* < 0.01).

Previously, we and others have characterized the biological factors associated with gene coexpression (see Iancu et al., 2010, 2012b); the strongest influences arise from shared TFBS, the existence of PPI between gene products, and the degree of spatial overlap between gene expression patterns. These factors were quantified for the edges in all networks. Genes with known PPI, the edge weights were significantly higher than for randomly selected gene pairs (Figure 5A). Gene pairs sharing TFBS had increased coexpression and CoSplicEx but not cosplicing (Figure 5B). The reverse was true for splicing factor binding sites SFBS. Cosplicing and CoSplicEx connectivity had a modest but statistically significant correlation with the number of SFBS within a gene. By following the evolutionary history of the network hubs, we found that cosplicing hubs show dramatic increases in number and length of introns, to a much bigger extent than other genes. While intron gain has been extensively studied (Babenko et al., 2004), considerable controversy persists as to the exact functional role of intronic sequences. If we accept the premise that network connectivity is related to functionality, our results suggest one possible explanation for the presence of highly conserved intronic sequences across many organisms. Based on the fact that large introns proportionally increase the number of SFBS, which in turn increase cosplicing connectivity, we hypothesize that long intronic sequences have been retained because of their role in splicing coordination, both by increasing the number of SFBS and lowering the GC content.

For genes sharing microRNA binding sites, coexpression was modestly increased but cosplicing and CoSplicEx was significantly decreased (*p* < 10^−15^) (Figure 5D). This result is consistent with one of the mechanisms by which microRNAs are thought to modulate transcription (Guo et al., 2010): a reduction in mRNA production the effects on cosplicing, which are manifested by reduced edge weights, are more difficult to interpret. While purely computational approaches cannot offer a definite explanation, we hypothesize that reduced cosplicing is consistent with another mechanism by which microRNAs are thought to affect transcription: a reduction of the stability of mRNA (Valencia-Sanchez et al., 2006). When two gene transcripts are simultaneously affected by microRNAs in this manner, their reduced stability might increase the uncorrelated variability in the exon counts, thus rendering the Canberra distances more variable and reducing the Mantel correlation.

Our distance measure approach has the advantage of utilizing most of the data generated in the RNA-Seq experiment but the disadvantage that the granularity associated with an exon-level analysis is not readily accessible. The data extracted allow one to conclude that isoform variability is coordinated between two genes but does not detect which isoform or isoforms contribute the most to this coordination. To some extent this problem is mitigated by the fact that for most genes there are relatively few and in a majority of cases only two highly expressed isoforms. Splicing is estimated to occur for 95% of all multiexon genes (Pan et al., 2008), 86% of which have a minor isoform that accounts for > 15% of total gene expression (Wang et al., 2008; Barrie et al., 2012).

In conclusion, our results demonstrate that gene splicing is a highly coordinated process that can be efficiently described using network and graph theory concepts. Coexpression and cosplicing appear to be generated by distinct biological factors and have complementary roles within the transcriptional system. Depending on the goals of the analysis, exon level data facilitates either a focused approach on coexpression/cosplicing, or a more comprehensive view that combines both types of gene interactions within the CoSplicEx network.

### Conflict of interest statement

The authors declare that the research was conducted in the absence of any commercial or financial relationships that could be construed as a potential conflict of interest.

## Footnotes

^1^Gentleman, R., and Falcon, S. microRNA: Data and Functions for Dealing with microRNAs. R package version 1.26.0.

## Supplementary material

Supplemental Table1 **Gene Ontology annotations for unique coexpression, unique cosplicing, and dual hubs**.
